# Low T3 syndrome and short-term outcomes in patients with acute decompensated heart failure: A retrospective observational study

**DOI:** 10.6026/973206300222112

**Published:** 2026-04-30

**Authors:** Noorussaba Arfeen, Devendra Kumar Sinha, Kaushal Kishore

**Affiliations:** 1 Department of General Medicine, Patna Medical College and Hospital (PMCH), Patna, Bihar, India

**Keywords:** Low triiodothyronine (T3) syndrome, acute decompensated heart failure (ADHF), short-term outcomes, in-hospital mortality, euthyroid sick syndrome

## Abstract

Acute decompensated heart failure (ADHF) drives frequent hospitalizations with high short-term morbidity/mortality, yet low triiodothyronine (T3) syndrome characterized by unexplained low serum free T3 remains understudied as a prognostic marker in resource- limited ADHF settings. Therefore, it is of interest to assess the prevalence of low T3 and its association with in-hospital mortality, length of stay and 30-day readmission in 115 ADHF patients. Low T3 syndrome affected 40.9% (47/115) of patients, who exhibited higher in- hospital mortality (25.5% versus 7.4%, p=0.006), longer stays (9.8±3.6 versus 6.9±2.4 days) and greater 30-day readmissions (34.3% versus 15.9%) than normal T3 controls. Multivariate analysis confirmed that low T3 was an independent predictor of mortality (adjusted OR 3.42, 95% CI 1.28-9.15, p=0.014) after adjusting for age, ejection fraction and creatinine. Thus, we show low T3 syndrome as a prevalent, actionable prognostic biomarker for risk stratification and targeted intervention in resource-constrained ADHF management.

## Background:

Heart failure is a worldwide health issue, causing a clinical challenge of about 64 million people all around the world and imposing a burden on healthcare systems due to their frequent hospitalizations and a high rate of mortality [1]. The rapid deterioration of signs and symptoms of heart failure, forcing a patient to seek emergency medical care, is known as acute decompensated heart failure, which takes up a considerable percentage of emergency department presentations and is a major cause of death in-hospital among cardiovascular diseases [2]. Although pharmacological and device-based treatment have advanced, the short-term outcome of ADHF is still poor, with in-hospital mortality rates of between 4 and 15 percent and 30-day readmission rates of over 20 percent being common in most populations [3]. This has led to a continuous demand for reliable, readily available biomarkers on short notice to enhance early risk stratification and clinical decision- making. The thyroid hormone axis is very important for cardiovascular homeostasis, as it regulates myocardial contractility, systemic vascular resistance, heart rate and cardiac output [4]. The thyroid hormone in its active form, triiodothyronine, has direct genomic and non-genomic effects on cardiomyocytes, which impact calcium regulation, mitochondrial activity and expression of contractile proteins [5]. Another adaptive pattern of thyroid alteration, termed euthyroid sick syndrome or non-thyroidal illness syndrome, is common in the acute illness setting and is characterized by reduced serum T3 levels, normal or low thyroid-stimulating hormone (TSH) levels and fluctuating thyroxine (T4) levels [6]. This compensatory, peripheral deiodination-mediated, cytokine-regulated and thyroid hormone-binding protein-mediated change in the hypothalamic-pituitary-thyroid axis was historically thought to be a protective response to a lower metabolic load in the face of critical illness [7]. There is growing evidence, however, to indicate that low T3 syndrome can be maladaptive about cardiovascular disease, especially in congestive heart failure.

It has been shown that decreased circulating T3 levels are linked to impaired myocardial functioning, augmented neurohormonal responsiveness and unfavorable hemodynamic measures in chronic heart failure patients [8]. Several prospective studies have shown that low fT3 levels have independent predictive value for mortality among hospitalized heart failure patients, even after adjustment for established prognostic factors such as left ventricular ejection fraction (LVEF), B-type natriuretic peptide (BNP) and renal function [9]. Also, T3 replacement therapy has been demonstrated to improve cardiac performance and mitigate pathological remodelling in animal models of heart failure, providing biological plausibility for the prognostic relevance of T3 deficiency [10]. Even with this accumulated evidence, several gaps remain in the existing literature. To begin with, most studies that look into the association between low T3 syndrome and the outcome of heart failure have been centered in Western and East Asian regions and there is limited information on other cohorts of South Asians, especially the Indian subcontinent [11]. Considering the peculiarities of the epidemiology of heart failure in India (younger age at onset, rheumatic heart disease and specific comorbidity patterns), it is not possible to assume the generalizability of the available evidence to the Indian population [12]. Second, a significant number of previous studies concentrated on chronic stable heart failure or mixed samples, as well as specific studies of low T3 syndrome in the acute decompensated environment, are rather limited [13]. Third, the relationship between low T3 syndrome and other known prognostic factors in ADHF, such as renal dysfunction and anemia, is an area that should be further investigated to establish the independent and additive prognostic value of thyroid hormone assessment [14]. Therefore, it is of interest to identify the prevalence of low T3 syndrome among patients admitted with ADHF at a tertiary care hospital in eastern India and to assess its correlation with short-term clinical outcomes, including in-hospital mortality, length of stay and 30-day readmission rates.

## Materials and Methods:

## Study design and setting:

It was a retrospective observational study conducted in the Department of Medicine at Patna Medical College and Hospital (PMCH), Patna, Bihar, India.

## Population and sample size of the study:

One hundred and fifty-five (115) patients who were hospitalized with the main diagnosis of ADHF throughout the period of study were considered in the analysis. The sample size was calculated based on the availability of complete medical records that met the inclusion criteria within the study period and a convenience sample was used.

## Inclusion criteria:

Patients had to fulfill the following criteria: (a) age 18 and older; (b) The Department of Medicine was the location of admission; (c) the primary diagnosis was ADHF according to the Framingham criteria; (d) echocardiographic evaluation was performed during the initial hospital admission; (e) complete medical records (demographic data, clinical data, laboratory data and outcome data) was available.

## Exclusion criteria:

The exclusion criteria were: (a) known preexisting thyroid disease (hypothyroidism, hyperthyroidism or thyroiditis) or receiving thyroid hormone replacement therapy or antithyroid medication; (b) thyroidectomy or radioiodine therapy; (c) use of drugs that have been shown to have a significant effect on thyroid activity (amiodarone, lithium, high doses of corticosteroid); (d) acute coronary syndrome as the initial cause of hospitalization; (e) end stage renal disease under dialysis;

## Data collection:

The hospital's electronic and paper-based medical records were used to extract data. The variables were as follows: demographic (age, sex), comorbidity (hypertension, diabetes mellitus, coronary artery disease, chronic kidney disease, atrial fibrillation, valvular heart disease), clinical (heart rate, systolic and diastolic blood pressure, respiratory rate, oxygen saturation, NYHA functional class), lab (serum fT3, fT4, TSH, hemoglobin, serum creatinine, blood urea nitrogen, serum sodium, serum potassium, serum albumin). The readmission data within 30 days were determined based on the hospital admission records and the telephonic follow-up records at the case files.

## Definition of low T3 Syndrome:

Low T3 syndrome was determined as a serum fT3 below the lower portion of the laboratory reference range (< 2.0 pg/mL) with normal or low TSH (< 10 mIU/L) and fT4 (within or below the normal range) level. The patients with a high level of TSH (> 10 mIU/L), which is indicative of primary hypothyroidism, were excluded from the study.

## Grouping:

Patients were split into two categories based on admission fT3 levels: Group A (low T3, ft3 < 2.0 pg/mL) and Group B (normal T3, ft3 2.0 pg/mL).

## Statistical analysis:

The SPSS version 26.0 (IBM Corp., Armonk, NY, USA) was used in data analysis. The means of the continuous variables were presented as means with standard deviations (SDs) and were tested using the independent samples t-test when the variables were normally distributed, or the Mann-Whitney U test when the variables were not normally distributed, as dictated by the Shapiro-Wilk test. The categorical variables were presented as frequencies and percentages and compared using the chi-square test or Fisher's exact test, depending on the situation. Univariate and multivariate binary logistic regression analyses were conducted to identify predictors of in-hospital mortality and the results were presented as odds ratios (ORs) with 95% confidence intervals (CIs). The information that had p < 0.10 in the univariate analysis was included in the multivariate model. Receiver operating characteristic (ROC) curve analysis was conducted to assess the ability of fT3 levels to predict in-hospital mortality and the area under the curve (AUC) is presented. All analyses were deemed to be statistically significant at a p-value of less than 0.05.

## Results:

Of the 115 patients included, 47 (40.9%) had low T3 syndrome (Group A) and 68 (59.1%) had normal T3 levels (Group B). The mean age of the entire cohort was 59.4 ± 12.7 years and 71 (61.7%) were male. Patients with low T3 syndrome were significantly older (63.8 ± 11.4 versus 56.3 ± 12.8 years, p = 0.002) and had a lower mean LVEF (28.6 ± 7.2% versus 33.4 ± 8.5%, p = 0.002). The prevalence of diabetes mellitus and chronic kidney disease was higher in Group A, though only the difference in chronic kidney disease was statistically significant. Baseline characteristics are summarized in Table 1 (see PDF). In-hospital mortality was significantly higher in the low T3 group compared to the normal T3 group (25.5% versus 7.4%, p = 0.006). The mean length of hospital stay was also significantly longer in Group A (9.8 ± 3.6 versus 6.9 ± 2.8 days, p < 0.001). The need for inotropic support (48.9% versus 27.9%, p = 0.021) and mechanical ventilation (21.3% versus 8.8%, p = 0.062) were both more frequent in patients with low T3 syndrome. However, the difference in mechanical ventilation use did not reach conventional statistical significance. Among patients who survived to discharge, the 30-day readmission rate was significantly higher in Group A (34.3% versus 15.9%, p = 0.024). Outcome data are presented in Table 2 (see PDF). On univariate logistic regression analysis, low T3 syndrome (OR 4.31, 95% CI 1.41-13.18, p = 0.010), age > 65 years (OR 2.87, 95% CI 1.02-8.09, p = 0.046), LVEF < 25% (OR 3.56, 95% CI 1.24-10.22, p = 0.018), serum creatinine > 1.5 mg/dL (OR 3.12, 95% CI 1.09-8.94, p = 0.034), serum sodium < 130 mEq/L (OR 2.94, 95% CI 1.01-8.56, p = 0.048) and systolic blood pressure < 100 mmHg (OR 3.78, 95% CI 1.30-10.98, p = 0.015) were significant predictors of in-hospital mortality. In multivariable analysis, adjusting for age, LVEF, serum creatinine, serum sodium and systolic blood pressure, low T3 syndrome remained an independent predictor of in-hospital mortality (adjusted OR 3.42, 95% CI 1.28-9.15, p = 0.014). Results are displayed in Table 3 (see PDF). ROC curve analysis demonstrated that serum fT3 at admission had moderate discriminative ability for predicting in-hospital mortality, with an AUC of 0.756 (95% CI 0.641-0.871, p = 0.001). The optimal cutoff value of fT3 for predicting mortality was 1.72 pg/mL, yielding a sensitivity of 76.5% and a specificity of 69.4%.

## Discussion:

The current paper shows that low T3 syndrome is very common among the patients hospitalized with ADHF, with a prevalence rate of about 41 percent in our sample and it is a definite independent predictor of the following outcomes: Poor short-term outcomes in the form of increased in-hospital mortality, extended hospital stay and increased readmission rates. These results underscore the clinical importance of thyroid hormone testing in acute heart failure and contribute to the growing body of evidence supporting fT3 as a prognostic biomarker in cardiovascular disease. Low T3 syndrome is widespread in this study, consistent with previous reports across different populations. The prevalence rate was reported to be about 30 percent in chronic heart failure patients in a large Italian multicenter study [15]. Although studies conducted in Chinese cohorts that were hospitalized with acute heart failure recorded a range of 35 to 58 percent prevalence rate [16]. This relatively high prevalence was probably due to the severity of the illness at the onset because most of the patients were in the NYHA functional class III or IV and a number of the patients had grossly impaired LVEF. The pathophysiology behind the decreased T3 in heart failure is multifactorial including, but not limited to, decreased hypothalamic-pituitary-thyroid axis activity of type 1 and type 2 iodothyronine deiodinases that are involved in peripheral T4 to T3 conversion, increased activity of type 3 deiodinase that degrades T3, as well as cytokine-induced depressions of hypothalamic-pituitary-thyroid axis activity and decreased thyroid hormone binding protein synthesis [17]. Our cohort results showing a low T3 association with higher in-hospital mortality are consistent with those of multiple previous studies. This has been demonstrated by a prospective study of 573 patients with ADHF, which showed that low fT3 has the strongest independent predictive strength for cardiac death, compared with BNP and LVEF [18]. Similarly, in a meta- analysis of more than 6,000 patients with heart failure, it was once again established that low T3 levels were linked to an almost two fold risk of all-cause mortality [19]. The independent prognostic value of low T3 syndrome for in-hospital mortality, as found in our multivariate analysis (adjusted odds ratio of 3.42), continues to support the notion that this parameter is independent of more conventional risk factors. The toxic effects of T3 deficiency in the failing heart may result from several mechanisms. At the cellular level, T3 controls the expression of major genes related to myocardial contractility, including the alpha-myosin heavy chain gene and sarcoplasmic reticulum calcium ATPase (SERCA2a), which are suppressed in T3-deficient conditions, leading to poor systolic and diastolic function [20]. T3 also regulates mitochondrial biogenesis and oxidative phosphorylation and its loss can augment the energetic deficiency of the impacted myocardium [21]. Also, low T3 levels have been linked to activation of the renin-angiotensin-aldosterone and sympathetic nervous systems, which favor maladaptive neurohormonal cascades that advance heart failure [22]. It is interesting to note that the systolic blood pressure of patients with low T3 syndrome was substantially lower at admission than the resting heart rate, which was higher than in the subgroup, indicating more advanced hemodynamic compromise in low T3 syndrome. It has been shown by the previous hemodynamic studies that low T3 level is associated with low cardiac output, high pulmonary capillary wedge pressure and high systemic vascular resistance in patients with heart failure [23]. The increased chronic kidney disease and reduced serum albumin in the low T3 group may also play a role in the negative outcomes that were witnessed, as both renal dysfunction and hypoalbuminemia are well- known predictors of poor prognosis in ADHF [24]. Nevertheless, despite the correction for serum creatinine, the independent relationship between low T3 and mortality remained, indicating that the prognostic effect of T3 deficiency is not confined to renal deficiency. The higher 30-day readmission rates in the low T3 group have significant clinical implications, as readmission in heart failure is a primary focus of quality improvement efforts and health care expense reduction. Although the mechanisms underlying this relationship require further research, it is reasonable to assume that a persistent T3 deficiency at discharge indicates incomplete recovery from the acute illness and that the patients will remain at risk of clinical deterioration. There is a recent study that has investigated serial fT3 levels in patients admitted with heart failure, which showed that when fT3 did not normalise at the time of discharge, this was linked to a three times higher death rate in 90 days [25]. The analysis of ROC curves in our study showed that variable fT3 had an AUC of 0.756 for predicting in-hospital mortality, indicating moderate discriminative ability. This can be compared with the prognostic performance reported for established biomarkers (NT-proBNP) in some ADHF populations and suggests that fT3 can be used to complement prognostic information [26]. The optimal cutoff of 1.72 pg/mL identified in our analysis is comparable to thresholds reported in other studies and may be used in practice as a decision point for risk stratification, but requires external validation in larger cohorts before adoption. One of the strengths of this study is that it adds to the limited information on low T3 syndrome in ADHF in the Indian subcontinent, where heart failure epidemiology differs from that of Western populations. The relationship between clinical outcomes and thyroid hormone abnormalities may be influenced by the younger age at which heart failure develops, the increased burden of rheumatic heart disease and socio-economic factors affecting access to healthcare in this region [27]. Our results indicate that the prognostic value of low T3 syndrome does not diminish in this clinical and geographic setting, underscoring the need to assess thyroid function in the management of ADHF. There are several shortcomings of this research worth noting. The retrospective design does not allow the development of causal relationships and can result in selection and information bias. The sample size, though sufficient to identify significant between-group differences in the primary outcome, might not have been adequate to detect differences in secondary outcomes, including mechanical ventilation use and to carry out more fine-grained subgroup analyses. Thyroid activity was evaluated only once on admission, not serially, which would have provided information on the course and management of the T3 abnormalities. NT-proBNP levels were not equally present across all patients, which prevented us from directly comparing the prognostic performance of fT3 with that of natriuretic peptides. Lastly, the findings can be generalized because the study was single-centered and the results can be validated in other centers. Prospective studies involving serial measurements of thyroid hormones should be conducted in the future, as they would help outline the time dependence of T3 dynamics and the clinical course of ADHF. The potential treatment effect of T3 supplementation in heart failure patients with low T3 syndrome is an interesting area of study and early-stage clinical trials have provided encouraging evidence of hemodynamic benefits [28].

## South Asians:

Multicenter studies with larger populations should be conducted to confirm the prognostic thresholds identified in this study and to establish whether the use of thyroid hormone to guide management interventions is likely to yield better clinical outcomes in ADHF.

## Conclusion:

Low T3 syndrome affected 41% of acute decompensated heart failure patients and strongly correlated with higher in-hospital mortality, prolonged stays, greater inotrope needs and 30-day readmissions. Initial serum fT3 levels provided moderate prognostic discrimination for mortality (cutoff 1.72 pg/mL with clinically relevant sensitivity/specificity). Thus, routine fT3 testing should be integrated into ADHF risk stratification to identify high-risk patients for enhanced monitoring and targeted interventions.
